# Draft Genome Sequence of *Fusobacterium nucleatum* subsp. *animalis* ChDC F324, Isolated from a Human Subgingival Plaque in the Republic of Korea

**DOI:** 10.1128/genomeA.01042-13

**Published:** 2013-12-12

**Authors:** Soon-Nang Park, Eugene Cho, Hwa-Sook Kim, Dae-Soo Kim, Jaeeun Jung, Jeong-Hun Baek, Yun Kyong Lim, Eojin Jo, Young-Hyo Chang, Jeong Hwan Shin, Sang-Haeng Choi, Jihee Kang, YongUn Choi, Si-Won Kong, Sang-Eun Han, Hong-Seog Park, Hongik Kim, Joong-Ki Kook

**Affiliations:** Korean Collection for Oral Microbiology and Department of Oral Biochemistry, School of Dentistry, Chosun University, Gwangju, Republic of Koreaa; Department of Dental Hygiene, Chunnam Techno University, Chunnam, Republic of Koreab; Human Derived Material Center, Korea Research Institute of Bioscience and Biotechnology (KRIBB), Daejeon, Republic of Koreac; Macrogen Inc., Gasan-dong, Seoul, Republic of Koread; Korean Collection for Type Cultures, Biological Resource Center, KRIBB, Daejeon, Republic of Koreae; Department of Laboratory Medicine, Busan Paik Hospital, Inje University College of Medicine, Busan, Republic of Koreaf; Research Institute of Unitech Science Co., Ltd., Daejeon, Republic of Koreag; SolGent Co., Ltd., Gwanpyeong-Dong, Daejeon, Republic of Koreah; R&D Division, Vitabio, Inc., Daejeon, Republic of Koreai

## Abstract

Five subspecies of *Fusobacterium nucleatum* have been classified: *animalis*, *nucleatum*, *polymorphum*, *vincentii*, and *fusiforme*. *F. nucleatum* subsp. *animalis* ChDC F324 (KCOM 1325) was isolated from a human subgingival plaque in the Republic of Korea. Here, we report the draft genome sequence of the strain.

## GENOME ANNOUNCEMENT

*Fusobacterium nucleatum* is a Gram-negative anaerobe. *F. nucleatum* might play an important role in the formation of dental plaque acting as a bridge between the early colonizers and late colonizers (1). Five subspecies of *Fusobacterium nucleatum* have been classified: *animalis*, *nucleatum*, *polymorphum*, *vincentii*, and *fusiforme* (2, 3). *F. nucleatum* subsp. *animalis* ChDC F324 (KCOM 1325) was isolated from a human subgingival plaque. In this report, we present the draft genome sequence of *F. nucleatum* ChDC F324.

Draft sequencing was performed by the Macrogen Co., (Seoul, South Korea) using the Illumina Hiseq 2000 system sequencing technology. We constructed 101 paired-end sequencing libraries with insert sizes of about 200 bp and generated 52,466,916 bp of usable sequence. We assembled the reads using SOAPdenovo (http://soap.genomics.org.cn). SOAPdenovo v. 1.05 was run with option K79 and configuration options reverse_seq of 0 (standard mate-pair orientation), asm_flags of 3 (try harder to build large contigs), and rank of 1 (reads were used while scaffolding). The assembled reads were assembled into 123 contigs with a size ranging from 208 to 121,709 bp (total 2,265,718 bp) and the GC content was 26.86%. Open reading frames were predicted and annotated using the Glimmer 3.02 modeling software package (4). The predicted protein sequences were annotated as Gene Ontology (GO) by the basic local alignment search tool (BLAST). Then, the GO classes were grouped into a total of 124 GO-Slim terms using the web tool CateGOrizer (5).

The genome contained 2,174 protein-coding genes, 35 tRNA genes, and several key pathways for amino acids, carbohydrates, lipids, and organic acids. Biosynthetic pathways exist for at least four amino acids, aspartate, asparagine, glutamate, and glutamine. The draft genome sequence contained virulence factors such as butyrate fermentation-related genes, hemolysin, zinc metalloprotease, RelE/StbE replicon stabilization toxin, serine protease, outer membrane lipoprotein (CmeC), macrolide export ATP-binding/permease protein (MacB), 5-nitroimidazole antibiotic resistance proteins, beta-lactamase, multidrug resistance proteins, multi-antimicrobial extrusion proteins, macrolide-efflux protein, outer membrane porin F, toxin YoeB, zeta toxin, and TonB protein. The genome contained the oxidative stress-response genes such as glutaredoxin, glutathione peroxidase, NADH oxidase, and rubrerythrin. The genome also contained the five two-component systems and one unmatched sensor histidine kinase.

### Nucleotide sequence accession numbers.

This whole-genome shotgun project has been deposited at DDBJ/EMBL/GenBank under the accession number ATKD00000000. The version described in this paper is the first version, ATKD01000000.
